# Modelling the dynamic vaccination game with evolutionary feedback: exploring pairwise interactions and vaccine strategies

**DOI:** 10.1098/rsos.240460

**Published:** 2024-06-19

**Authors:** Khondoker Nazmoon Nabi, K. M. Ariful Kabir

**Affiliations:** ^1^ Department of Mathematics, Bangladesh University of Engineering and Technology, Dhaka 1000, Bangladesh

**Keywords:** feedback-evolving game, evolutionary game theory, socio-economic cost, environmental dynamics

## Abstract

A novel approach rooted in co-evolutionary game theory has been introduced to investigate how the interaction between human decision-making and the dynamics of the epidemic environment can shape vaccine acceptance during disease outbreaks. This innovative framework combines two key game concepts: the cooperation–defection game and the cost–benefit vaccination game. By doing so, it enables us to delve into the various factors that influence the success of a vaccination campaign amid an outbreak. Within this framework, individuals engage in a thorough evaluation of the risks, benefits and incentives associated with either cooperating by getting vaccinated or defecting by refusing the vaccine. Additionally, it involves a careful analysis of the costs and benefits linked to vaccine acceptance. The outcomes of this study stress the importance of two main factors: the effectiveness of the vaccine and the prevalence of a cooperative culture within society. This insight into the strategic interactions between individuals and their decisions about vaccination holds significant implications for public health policymakers. It equips to boost vaccination coverage and address vaccine hesitancy within society ultimately contributing to better public health outcomes during epidemic outbreaks.

## 1. Introduction

Understanding human behaviours in an epidemic outbreak setting has always been an intricate challenge for public health experts and policymakers [1,2]. Considering outbreak severity and resource availability, they need to develop effective public health intervention policies to control the disease outbreak and mitigate public suffering. Vaccination is one of the most effective intervention policies that contribute to establishing herd immunity in the population, reducing disease transmission and limiting the spread of any infectious disease in any community. The success of any vaccination programme depends on different underlying factors that influence individuals’ decision-making processes regarding vaccine acceptance [3–6]. These factors include limiting the spread of any infectious disease in any community, cost of vaccination, vaccine efficacy, vaccine dilemma, infection risk and levels of cooperation in society [7–10]. High levels of human cooperation in society result in a higher vaccination uptake, leading to broader community protection and reduced transmission [11]. In addition, by fostering a sense of shared responsibility and emphasizing the collective welfare of vaccination, human cooperation can significantly shape vaccine acceptance behaviours, which can contribute to effective epidemic control and desired public health outcomes. In contrast, overcoming vaccine hesitancy has constantly challenged public health policymakers [12,13]. Researchers have been relentlessly designing a theoretical framework to address vaccine scepticism in the population.

Human cooperative behaviour is influenced by the outcomes of a pairwise game, where interactions occur between two players with two possible strategies: cooperation and defection [14,15]. Depending on their cooperative or defective preferences, players receive different pay-offs. If both players cooperate they receive a reward (R), and a punishment (P) if both defect. Additionally, there are the sucker (S) and temptation (T) pay-offs for one player cooperating and the other defecting, respectively. The game can be characterized by two parameters, $D_{g}$ and $D_{r}$ (the gamble-intending dilemma and the risk-averting dilemma), which represent the rescaled properties of the universal dilemma strength [16–18]. The magnitude of $D_{g}$ and $D_{r}$ determines the classification of the game into four classes: trivial ( $D_{g}<0$ and $D_{r}>0$ ) with no dilemma, prisoner’s dilemma ( $D_{g}>0$ and $D_{r}>0$ ), chicken ( $D_{g}>0$ and $D_{r}<0$ ) and stag hunt ( $D_{r}>0$ and $D_{g}<0$ ). Much of the existing research in pairwise game theory focuses on dilemma strength (DS). Therefore, the cooperative behaviour of humans is influenced by the outcomes of pairwise games, where different pay-off structures and parameters determine the type and strength of the dilemmas players face. Extensive research has been conducted to study the impact of dilemma strength on cooperative decision-making.

Liu *et al*. [19] studied a co-evolutionary game dynamics framework to understand how the complex interplay of competitive cognitions and public opinion environment leads to the emergence of different cognitive strategies and diverse public opinion profiles. Weitz *et al*. [20] studied how feedback mechanisms between individual decision-making and environmental conditions can lead to oscillatory resource exploitation and regeneration behaviour, resulting in periodic collapses and regeneration of resource stock. Several recent studies [21–25] provide insights into the evolution of vaccine acceptance in a cooperative environment in the presence of free-riders and vaccine sceptics. Competitive cognition dynamics can simulate situations where individuals face conflicting cognitions, such as public health issues, social polarization, pro-vaccination [26], anti-vaccination [26], social distancing [27], mask-wearing [28], truth and rumours. This aspect of conflict has been explored in the infectious disease field to understand competitive diffusion during co-contagion processes [29]. Moreover, the state of the environment, whether it is abundant or depleted, can influence individual preferences. As the environment degrades, there is an increased tendency towards cooperation. Several recent studies have theoretically investigated this idea [30–32], examining the uncertainty of shared resources in eco-evolutionary dynamics using evolutionary game theory. These studies explore strategic relationships between cooperators and defectors. Considering the above literature review, this article combines the concepts of the vaccination game and pairwise game to examine the feedback between games and the environment in an epidemic disease model. The behavioural aspect surrounding individuals constitutes the population’s environment. Therefore, this research focuses on the feedback between game dynamics and the environment, considering the evolutionary nature of vaccination and pairwise games.

In this study, we introduce a co-evolutionary game-theoretic model structure integrating dyadic games and vaccination cost–benefit games incorporating game-environment feedback to have insights into vaccination behaviours, vaccine dilemmas and effective disease control policies. This framework allows us to analyse different factors for vaccine acceptance, community cooperation, conditional cooperation, free-riding and defection on the local time scale. Our study shows that the influence of feedback mechanisms in strategic interactions is crucial for promoting vaccine acceptance in the community.

## 2. Model and methods

In this section, we introduce a framework of an evolutionary game-theoretic model incorporating game-environment feedback that has been designed in this study, where we combine dyadic games with vaccine cost–benefit games in an epidemic outbreak setting to gain a deeper understanding of vaccine uptake behaviours and human decision-making processes to curb the spread of disease. This framework allows us to investigate different underlying factors of updating individuals' strategies regarding whether to engage in vaccination programmes. Our study categorizes individuals into two broader groups: strategists and strategic vaccinators. Strategists carefully analyse the complex interplay of cooperation and defection in society. They evaluate the risks, benefits and incentives associated with cooperation and defection, considering the society’s personal and long-term collective welfare. After evaluating all the trade-offs, they try to adapt their strategies accordingly. On the other hand, another group of people, namely strategic vaccinators, evaluate risks, benefits and potential outcomes related to vaccination strategy and make their decisions accordingly.

### 2.1. Epidemic dynamics

**Figure 1.**
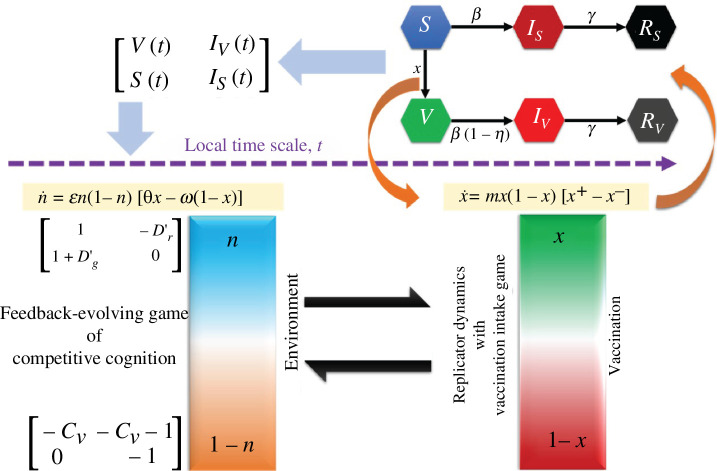
The epidemic model is depicted through a schematic diagram, portraying the population categorized into six distinct states: susceptible (S) , vaccinated $\left(V\right)$ , infected from susceptible individuals $\left(I_{S}\right)$ , infected from vaccinated individuals $\left(I_{V}\right)$ , recovered from susceptible infection $\left(R_{S}\right)$ and recovered from vaccinated infection $\left(R_{V}\right)$ . This model is designed to be applicable specifically during epidemic seasons and is analysed on a local time scale. The epidemic dynamics are intricately linked to the population’s behavioural traits, represented by the fraction of cooperators $\left(x\right)$ and defectors $\left(1-x\right)$ . These behavioural dynamics are modelled within the evolutionary game theory (EGT) framework, which explores how strategic interactions between individuals influence the spread of the epidemic. Within the EGT framework, the epidemic dynamics are further influenced by two distinct game scenarios: the vaccination game and the pairwise game. These games are governed by a shared resource $\left(n\right)$ , whose status undergoes periodic changes. An environmental feedback mechanism orchestrates the transition between the vaccination game and the pairwise game, creating an evolving game environment. The evolutionary behavioural game model considers both the vaccination and pairwise games simultaneously, operating on the same local time scale. This holistic approach allows for the comprehensive analysis of how individual strategies evolve in response to the changing dynamics of the epidemic. Furthermore, periodic effects are integrated into the model, capturing the cyclic nature of the epidemic and its impact on population behaviour.


A classical compartmental model SVIR (S—susceptible class, V—vaccinated, I—infected class and R—recovered class) [33,34] understands the transmission dynamics of any infectious disease outbreak in the population. Susceptible (unvaccinated) individuals are vulnerable to getting infected and do not have sufficient immunity to protect themselves. Susceptible individuals are susceptible to infection at a transmission rate of β (per person/day). Those vaccinated with a fully effective vaccine gain long-lasting immunity against the targeted infectious disease. However, individuals vaccinated with leaky vaccines experience reduced protection and may contract the disease. The vaccination rate is denoted as x , and the vaccine’s efficacy is represented by η . Individuals vaccinated with leaky vaccines can become infected at a rate of β(1-η) if they come into contact with either unvaccinated infected individuals $\left(I_{S}\right)$ or vaccinated individuals with leaky vaccines $\left(I_{V}\right)$ . Recovery from infection occurs at a rate of γ (per day) for individuals who have been infected $\left(I_{S}(t),I_{V}(t)\right)$ . Finally, $R_{S}(t)$ and $R_{V}(t)$ denote individuals who have recovered from infection among susceptible and vaccinated individuals, respectively. Vaccinated people who received leaky vaccine have partial protection against the disease. Both unvaccinated-infected and vaccinated-infected get recovered from the disease with recovery rate γ . The schematic diagram of this transmission flow of the infectious disease is illustrated in figure 1. The following system of ordinary differential equations based on compartmental mean-field approximation represents the transmission dynamics of the disease.

(2.1) $$\overset{˙}{S}=-\beta S\left(I_{S}+I_{V}\right)-xS,$$

(2.2) $$\dot{V}=xS-\beta \left(1-\eta \right)V\left(I_{S}+I_{V}\right),$$

(2.3) $$\dot{I_{S}}=\beta S\left(I_{S}+I_{V}\right)-{\gamma I}_{S},$$

(2.4) $$\dot{I_{V}}=\beta \left(1-\eta \right)V\left(I_{S}+I_{V}\right)-{\gamma I}_{V},$$

(2.5) $$\dot{R_{S}}={\gamma I}_{S},$$

(2.6) $$\dot{R_{V}}={\gamma I}_{V}.$$

### 2.2. Evolutionary dynamics

We investigate two symmetric games: pairwise interactions characterized by dilemma strength and vaccination involving associated costs. The pay-offs within these games are influenced by the supportive level of two cognitive strategies, which we denote as ‘game cognition’ and ‘vaccine cognition’, expressed through an environment-dependent pay-off matrix. These games form the foundation for applying co-evolutionary game theory to analyse the competition between these cognitive strategies. We categorize individuals into two classes: vaccinated (V) and unvaccinated/non-vaccinated (NV) . Additionally, based on the health status of individuals at the time, we further classify them into two groups: healthy (H) , representing those who are no longer infected and infected (I) .

Let us begin by considering the first symmetric two-player two-strategy game, which presents a dilemma characterized by the dilemma strengths (DS) of $D_{g}^{\prime }$ and $D_{r}^{\prime }$ , as outlined in table 1. In this game, individuals who remain healthy after participating in the vaccine programme receive a pay-off of 1, while those who become infected and are not vaccinated receive a pay-off of 0. Conversely, vaccinated but infected individuals receive a pay-off of $-D_{r}^{\prime }$ and non-vaccinated but healthy receive a pay-off of $1+D_{g}^{\prime }$ . Next, we incorporate a game structure representing the fraction of individuals obtained from the epidemic dynamics described by equations (2.1)–(2.6) in table 2. At any given time, t , the population is divided into four categories: healthy and vaccinated (V(t)) , healthy and non-vaccinated (susceptible, S(t) ), infected and vaccinated ( $I_{V}(t)$ ) and infected and non-vaccinated ( $I_{S}(t)$ ). Finally, we introduce another symmetric game that integrates a vaccination-based behavioural structure, as depicted in table 3. This game includes costs associated with vaccination $\left(-C_{v}\right)$ and infection $\left({-C}_{i}=-1\right)$ . Fortunately, individuals who remain non-vaccinated and stay healthy can avoid costs, while those who become infected incur a cost of $-C_{i}=-1$ . If $-C_{v}$ represents the cost of vaccination, then the cost $-C_{v}-1$ refers to individuals who, unfortunately, are infected despite either receiving the vaccination or defending against contagion. The feedback-evolving games pay-off matrix can be expressed as shown in tables 1–3.

**Table 1.** Pay-off structure for the pairwise game.

	*H*	*I*
***V***	1	$-D_{r}^{\prime }$
***NV***	$1+D_{g}^{\prime }$	0

**Table 2.** Pay-off structure for the fractions of individuals.

	*H*	*I*
***V***	V(t)	$I_{V}(t)$
***NV***	S(t)	$I_{S}(t)$

**Table 3.** Pay-off structure for the vaccine cost–benefit pay-off.

	*H*	*I*
***V***	$-C_{v}$	$-C_{v}-1$
***NV***	0	-1

In the presence of two cognitions, individuals prefer supporting the game cognition (cooperative behaviour) or the vaccine cognition (vaccine behaviour). The player’s pay-off or loss during the strategy updating process depends on the level of support for the two cognitions from the external environment to capture the evolving game dynamics influenced by feedback. This matrix represents the pay-offs concerning the supportive level of the two cognitions: pairwise game cognition, $G_{P}$ , and vaccine cognition, $G_{V}$ .


(2.7) $$G_{P}=\left(\begin{array}{cc} 1 & -D_{r}^{\prime }\\ 1+D_{g}^{\prime } & 0 \end{array}\right),$$

(2.8) $$G_{V}=\left(\begin{array}{cc} -C_{v} & -C_{v}-1\\ 0 & -1 \end{array}\right),$$

(2.9) $$G=nG_{P}+\left(1-n\right)G_{V},$$

(2.10) $$G=n\left(\begin{array}{cc} 1 & -D_{r}^{\prime }\\ 1+D_{g}^{\prime } & 0 \end{array}\right)+\left(1-n\right)\left(\begin{array}{cc} -C_{v} & -C_{v}-1\\ 0 & -1 \end{array}\right),$$

where n and (1-n) denote the contexts for predicting, respectively, game cognition and vaccination cognition. The expected benefits (fitness) of defectors (non-vaccinated) and cooperators (vaccinated) are


(2.11) $$x^{+}=\left\{V\left(t\right)\left[n-C_{v}\left(1-n\right)\right]+I_{V}\left(t\right)\left[\left(-D_{r}^{\prime }\right)n+\left(1-n\right)\left(-1-C_{v}\right)\right]\right\}/\left(V\left(t\right)+I_{V}\left(t\right)\right),$$

(2.12) $$x^{-}=\left\{S\left(t\right)\left(1+D_{g}^{\prime }\right)n-I_{S}\left(t\right)\left(1-n\right)\right\}/\left(S\left(t\right)+I_{S}\left(t\right)\right).$$

The average social pay-off (ASP),


(2.13) $$ASP=x^{+}+x^{-}.$$

Finally, according to the expected pay-off of strategists and strategic vaccinators ( $x^{+}$ and $x^{-}$ ), the well-known replicator equations can be written as follows,


(2.14) $$\overset{˙}{x}=mx\left(1-x\right)\left[x^{+}-x^{-}\right].$$

The parameter ‘ m ’ denotes the constant factor adjusting individual attributes with corresponding rates.

### 2.3. Replicator dynamics with feedback-evolving games

The state of the cognitive behaviour in the society is characterized by the scalar value n , and the term $n\left(1-n\right)$ ensures that the state of the cognitive behaviour is bounded by [0,1] . When n approaches 1 , the number of strategists increases compared with strategic vaccinators. In this situation, most of the people in society adapt their strategies based on the risk–benefit evaluation associated with cooperation–defection choices. However, if n approaches 0 , then the number of the strategic vaccinators becomes high. In this context, individuals do cost–benefit analysis associated with vaccination uptake strategy and take their decisions accordingly. A modified version of standard replicator dynamics where $f\left(x\right)=\theta x-\omega (1-x)$ describes the feedback of strategies with the state of the cognitive behaviour in the society. The term θ>0 is considered as the enhancing factor of behavioural strategists in the society, whereas the term ω>0 is considered as the boosting factor of strategic vaccinators in the society. The pay-off matrix G is dependent on the state of the cognitive behaviour in the society. Depending on the actions of strategists and strategic vaccinators, the state of cognitive behaviour changes in society. The dimensionless quantity ϵ describes the relative speed of changing the state of cognitive behaviour in the society compared with the change in the frequency of rational actors (strategists or strategic vaccinators).


(2.15) $$\overset{˙}{n}=\epsilon n\left(1-n\right)\left[\theta x-\omega \left(1-x\right)\right].$$

Equation (2.15) is used in evolutionary game theory to model the evolution of strategies or traits in a population of interacting agents. Here, ϵ is a parameter representing the intensity of selection or the strength of evolutionary pressure. The term (1-n) represents the proportion of the population that does not possess the trait. This factor contributes to the decrease in the trait frequency when n is high. (1-x) represents the proportion of individuals who do not adopt the strategy x . This factor contributes to the decrease in the trait frequency when x is high. θx represents the benefit or pay-off individuals receive for possessing the trait x . This term contributes positively to the growth of the trait when x is high. Finally, ω(1-x) represents the cost or disadvantage of not possessing the trait x . This term contributes negatively to the growth of the trait when x is low. Thus, the equation describes how the frequency of a trait n changes over time based on the interplay between selection pressure (ϵ) , the relative fitness of the trait (θx) and the cost of not possessing the trait (ω(1-x)) . It captures how traits or strategies spread or decline within a population over successive generations owing to evolutionary pressures.

## 3. Results and discussion

In this study, we investigate how the dynamics of game-environment feedback influence the development of cooperative behaviour (dyadic game) and vaccine uptake behaviours (cost–benefit game) among individuals during an epidemic outbreak setting on local time scale [35]. In this framework, our objective is to gain a deeper understanding of human decision-making in a vaccine-preventable disease outbreak setting. We explored a co-evolutionary theoretical analysis of the strategy-environment system under all possible circumstances. Our analysis focuses on the detailed dynamical patterns where there exist stable, unstable and saddle points. In such a situation, the system has five potential fixed points, with four located at the corners and one located as an interior fixed point. The Appendix contains a thorough theoretical analysis that explores the conditions for the existence of these fixed points, as well as their respective stability properties. We also investigate the impact of vaccine efficacy, vaccine dilemma, evolution of cooperation in cooperative-dominant (C-dominant or trivial) game and defection-dominant (D-dominant or prisoner dilemma) game settings. To achieve this, we conduct an analysis of different insightful metrics—final epidemic size (FES), vaccination coverage (VC), vaccine volunteers (VV) and average social pay-off (ASP) for varying state of cognitive behaviour in the society (n) , vaccine effectiveness (η) , vaccination cost ( $C_{v})$ ) in two different dyadic game settings (trivial and prisoner dilemma).

**Figure 2.**
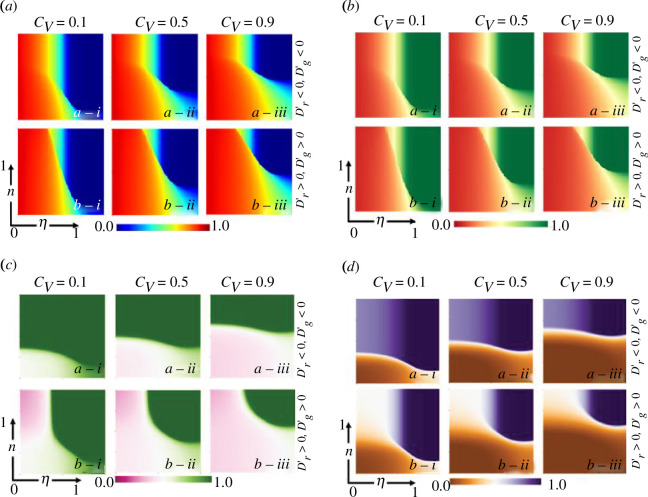
Two-dimensional phase diagrams of final epidemic size (*a*), vaccination coverage (VC) (*b*), vaccine voluntarists (VV) (*c*) and average social pay-off (ASP) (*d*) for both trivial ( $D_{r}^{\prime }<0,D_{g}^{\prime }<0$ ) and prisoner dilemma game ( $D_{r}^{\prime }>0,D_{g}^{\prime }>0$ ) along the state of the cognitive behaviour in the society $\left(n\right)$ and vaccine effectiveness $\left(\eta \right)$ considering three different vaccination cost settings ( $C_{v}=0.1,0.5\;\text{and}\;0.9$ ). Here, the state of cognitive behaviour (*n*) is considered as a parameter (not a state variable) by following equation (2.14).

A series of two-dimensional heatmaps (figure 2) provides insights into how individuals’ strategies change, as different key metrics-final epidemic size (panel *a*), vaccination coverage (panel *b*), vaccine volunteers (panel *c*) and average social pay-off (panel *d*) vary along vaccine effectiveness (η) and state of the cognitive behaviour in the society (n) in an epidemic outbreak setting. In this context, we regard the cognitive behaviour state within the society, denoted as n , as a parameter rather than a state variable by equation (2.11). We explore the range of n values from 0 to 1 to investigate the effects of various constant cognitive behaviours on altering epidemic dynamics and behavioural dynamics. By examining this spectrum of cognitive behaviour constants, we aim to elucidate how different cognitive behaviour settings influence the evolution of the epidemic and behavioural dynamics within society. Blocks (*b*)(*a*-i) and (*b*)(*b*-i) illustrate that if the cost of the vaccination is low and efficacy is high, individuals realize the collective benefits of vaccination (ASP is high) and start participating in vaccination programmes spontaneously. In consequence, high vaccination coverage is achieved which plays a crucial role in curbing the spread of the disease and reducing the FES (blocks (*a*)(*a*-i) and (*a*)(*b*-i)). In contrast, when the cost of vaccination ( $C_{v})$ becomes higher, individuals feel less motivated to get vaccinated which leads to lower vaccination coverage (blocks (*b*)(*a*-iii) and (*b*)(*b*-iii)) and potentially higher FES (blocks (*a*)(*a*-iii) and (*a*)(*b*-iii)). Blocks (*d*)(*a*-i) and (*d*)(*b*-i) highlight the fact that average social pay-off is relatively high in C-dominant society compared with D-dominant society, indicating spontaneous participation in vaccination programmes owing to the collective benefits of vaccination strategy over the potential risks associated with vaccination. As a result, the number of vaccine voluntarists is higher in cooperative society (blocks (*c*)(*a*-i, ii, iii)) as people prioritize collective well-being and realize the overall benefits of vaccines in protecting their own health and others, which contributes to the broader public health goals of curbing the disease spread and achieving herd immunity.

**Figure 3.**
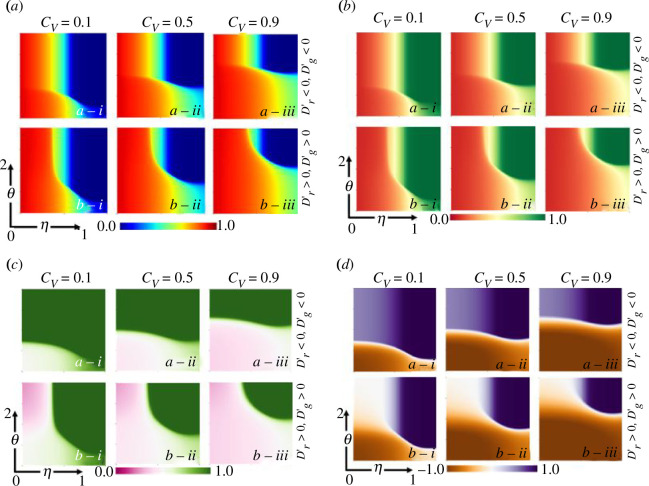
Two-dimensional phase diagram of final epidemic size (*a*), vaccination coverage (VC) (*b*), vaccine volunteers (VV) (*c*) and average social pay-off (ASP) (*d*) for both (*a*-*) trivial ( $D_{r}^{\prime }<0,D_{g}^{\prime }<0$ ) and (*b*-*) prisoner dilemma game ( $D_{r}^{\prime }>0,D_{g}^{\prime }>0$ ) along ratio of the growth rates to decline rates of strategists and strategic vaccinators $\left(\theta \right)$ and vaccine effectiveness (0≤η≤1) considering three different vaccination cost settings ( $C_{v}=0.1,0.5\;\text{and}\;0.9$ ).

The study includes another series of two-dimensional heatmaps (figure 3) to explore how feedback from the game environment influences human decision-making processes. These heatmaps depict the dynamics of various factors, such as the final epidemic size (panel *a*), vaccination coverage (panel *b*), vaccine volunteers (panel *c*) and average social pay-off (panel *d*). The heatmaps vary based on the effectiveness of the vaccine (*η*) and the ratio of growth rates to decline rates of strategists and strategic vaccinators (θ) . Blocks (*d*)(*a*-*) and (*d*)(b-*) specifically highlight that in a cooperative feedback-evolving society (θ>1) , the ASP tends to be higher. This is because these societies prioritize collective welfare and promote prosocial behaviour. As a result, individuals in these societies are more encouraged to participate in vaccine programmes voluntarily. In societies where a culture of cooperation prevails (blocks (*c*)(*a*-))*,* there is a noticeable increase in the number of vaccine volunteers compared with societies dominated by non-cooperation (blocks (*c*)(*b*-)). This trend of fostering a culture of collective action plays a crucial role in effectively controlling disease outbreaks.

Therefore, the outcomes demonstrate how the feedback from the game environment can shape human decision-making processes. Cooperative societies with a higher ASP value and a culture of collective action tend to exhibit higher levels of vaccination coverage and voluntary participation in vaccine programmes, ultimately contributing to better control of disease outbreaks.

**Figure 4.**
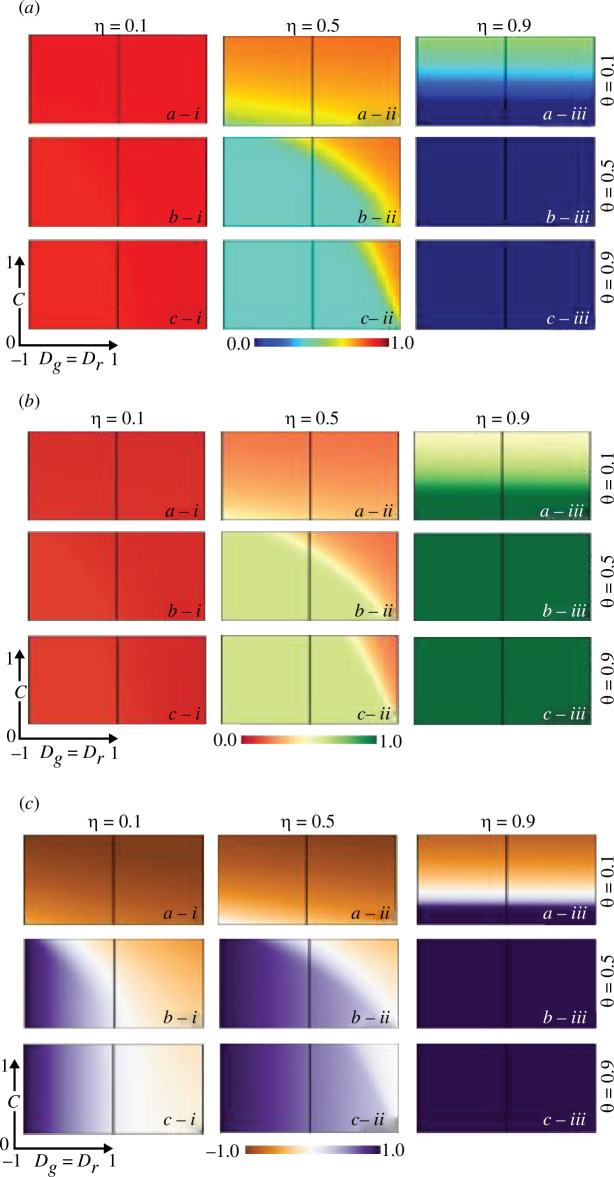
Two-dimensional phase diagram of final epidemic size (*a*), vaccination coverage (VC) (*b*) and average social pay-off (ASP) (*c*) along dilemma strength $\left(-1\leq D_{r}^{\prime }=D_{g}^{\prime }\leq 1\right)$ and cost of vaccination $\left({0\leq C}_{v}\leq 1\right)$ considering three different vaccination efficacy settings ( η=0.1,0.5and0.9 ).

Figure 4 provides insightful information on how individuals' strategies adapt in response to variations in crucial metrics during an epidemic outbreak. The figure consists of three panels: panel (*a*) represents the final epidemic size, panel (*b*) indicates the vaccination coverage and panel (*c*) depicts the average social pay-off. In blocks (*b*)(*a*-iii), (*b*)(*b*-iii) and (*b*)(*c*-iii), it is observed that when the cost of vaccination is low, and the vaccine efficacy is high (η=0.9) , individuals recognize the collective benefits of vaccination, resulting in a high ASP. Consequently, they spontaneously participate in vaccination programmes until a moderate culture of cooperation (θ≥0.5) is established within the society. When the cost of vaccination increases $\left(C_{v}=0.5\right)$ and the vaccine efficacy is moderate (η=0.5) , the evolution of cooperation becomes crucial for effectively controlling the disease outbreak (blocks (*b*)(*a*-ii), (*b*)(*b*-ii) and (*b*)(*c*-ii)). Individuals must adopt cooperative strategies to control disease in such scenarios successfully. On the other hand, blocks (*a*)(*a*-i), (*a*)(*b*-i) and (*a*)(*c*-i) depict a situation where the vaccine efficacy is low (η=0.1) . Individuals are discouraged from vaccinating in these cases, leading to low vaccination coverage and a high FES. Consequently, controlling disease outbreaks becomes highly challenging. Thus, figure 4 provides valuable insights into how individuals’ strategies are influenced by various vital metrics, such as the cost of vaccination, vaccine efficacy, dilemma strength and their impact on the final epidemic size, vaccination coverage and average social pay-off. It highlights the importance of high vaccine efficacy, low vaccination cost and the evolution of cooperation in effectively controlling disease outbreaks and minimizing their impact.

**Figure 5.**
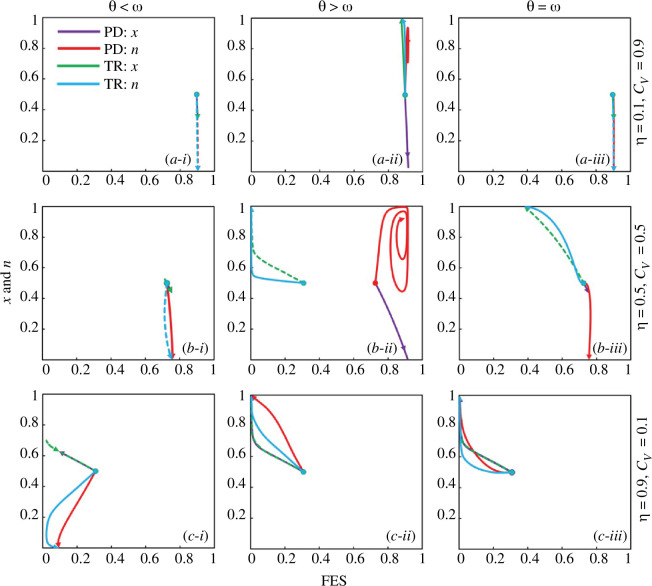
Trajectory graph of state of the cognitive behaviour in the society $\left(n\right)$ and the cooperation fraction ( x ) for both trivial (C-dominant) and prisoner dilemma (D-dominant) game along with final epidemic size (FES) for three different sets of values of vaccine efficacy and cost, (*a*-*) $\left(\eta =0.1,C_{v}=0.9\right)$ , (*b*-*) $\left(\eta =0.5,C_{v}=0.5\right)$ and (*c*-*) ( $\eta =0.9,C_{v}=0.1,$ respectively. Also, panels (*-i), (*-ii) and (*-iii) present for θ<ω , θ>ω, and θ=ω . The solid purple, red, green and blue lines mean trajectory path for x of PD, n of PD, x of TR and n of TR, respectively. Related parameters are β=0.83333 and γ=0.3333 .

In the above analysis, we concentrated on evolutionary games related to vaccination behaviour, specifically studying whether individuals opt for vaccination, considering either a fixed or changing ‘ n ’ value at equilibrium. However, this approach should have considered the potential for individuals to adapt their strategies in response to the evolving environment. To delve deeper into this aspect, we introduce dynamic and time-dependent environmental evolution dynamics in a time-dependent scenario, as depicted in figure 5.

Figure 5 depicts the feedback-driven mechanism that explores trajectories for variables ‘ n ’ and ‘ x ’ in relation to the FES. The panels (*-i), (*-ii) and (*-iii) correspond to situations where θ is less than ω , θ is greater than ω and θ is equal to ω , respectively. On the other hand, panels (*a*-*), (b-*) and (*c*-*) showcase the interplay between vaccine efficacy and vaccination cost, specifically for the scenarios where η (vaccine efficacy) is 0.1 and $C_{v}$ (vaccination cost) is 0.9, η=0.5 , $C_{v}=0.5$ and η=0.9 , $C_{v}=0.1$ , considering both PD (prisoner’s) and TR (trivial) cases. In panels (*-i) for θ<ω , it is noticeable that lower FES values are observed when vaccine efficacy is higher and vaccination cost is lower, as depicted in panel (*c*-i). Consequently, in this case of θ<ω , both PD and TR tend to converge towards 0. For θ>ω in panels (*-ii), we observe that ‘ n ’ exhibits a higher tendency, regardless of variations in vaccine cost and efficacy. Notably, panels (*a*-ii) and (*b*-ii) exhibit cyclic patterns in ‘ n ’. This cyclic behaviour is attributed to the feedback-driven mechanism when θ>ω , especially when the vaccine cost is less reliable and higher. This suggests that, in situations of lower reliability and higher cost, society faces a dilemma. We find a similar trend in panel (*a*-i) for θ=ω , like panel (*a*-iii). For the case of θ=ω , we observe that the ‘ n ’ values tend to approach 1 in panel (*b*-ii) for intermediate vaccine efficacy and cost. In panel (*c*-iii), when η=0.1 and $C_{v}=0.9$ , PD and TR tend to approach 1.

Furthermore, figure 5 highlights a key observation: when vaccine efficacy is lower, and vaccination cost is higher, the feedback evolving system consistently registers higher values, regardless of whether the society cooperates or opts for defection. However, in scenarios where vaccine efficacy and cost are moderate, cooperative behaviour within society becomes a critical factor in determining the success of the immunization programme. In situations with more strategists than strategic vaccinators ( θ>ω ) within the society, a significant decline in FES is evident when the society cooperates. Conversely, FES tends to increase when defection prevails within the society. In societies dominated by defection (D-dominant), the phase plane exhibits the presence of limit cycle orbits. Introducing an affordable vaccine with high efficacy can play a pivotal role in reducing FES, potentially leading to the eventual containment of the epidemic.

## 4. Conclusion

In conclusion, this study presents a novel co-evolutionary game-theoretic framework that provides valuable insights into the complex dynamics of vaccine acceptance during epidemic outbreaks. By integrating dyadic games and vaccination cost–benefit games with game-environment feedback, our research sheds light on the factors that influence vaccination behaviours and the effectiveness of disease control policies. Our findings underscore the significance of high vaccine efficacy, low vaccination costs and the evolution of cooperation in promoting vaccine acceptance and minimizing the impact of disease outbreaks. Furthermore, we emphasize the role of societal culture and the importance of fostering a culture of collective action in effectively controlling epidemics. Notably, feedback mechanisms within strategic interactions play a crucial role in shaping human decision-making processes, underscoring the need for dynamic and time-dependent environmental evolution dynamics in understanding these phenomena. In particular, the study highlights how the roll-out of affordable, highly effective vaccines can significantly reduce the burden of disease outbreaks, potentially leading to their containment.

The study’s key findings are centred on exploring adaptability in vaccination behaviour by introducing dynamic environmental evolution dynamics. It was obtained that lower vaccine efficacy and higher vaccination cost consistently resulted in elevated FES values, irrespective of societal cooperation or defection. In contrast, situations with moderate vaccine efficacy and cost emphasized the critical role of cooperative behaviour in achieving successful immunization programmes. Notably, when societies had more strategists than strategic vaccinators ( θ>ω ), FES decreased with cooperation but increased with defection. D-dominant societies exhibited limit cycle orbits, suggesting oscillatory behaviour. Introducing affordable, highly effective vaccines emerged as a critical strategy in significantly reducing FES and potentially bringing about the end of epidemics.

Overall, this research provides valuable insights for public health policymakers, offering a framework to design more effective intervention policies, enhance vaccination coverage and address vaccine hesitancy within society, ultimately contributing to better disease control and prevention.

## Data Availability

The datasets used and/or analysed during the current study are available online [36].

## Appendix A

### Stability of fixed points in dynamical model

The differential equations that represent the evolutionary system can be expressed as follows:

(A 1)
$$\left\{ \begin{array}{l} \overset{˙}{x}=mx\left(1-x\right)\left[x^{+}-x^{-}\right]\\ \overset{˙}{n}=\epsilon n\left(1-n\right)\left[\theta x-\omega \left(1-x\right)\right] \end{array}\right..$$

Here,

$$x^{+}=\frac{V\left(t\right)\left[n-C_{v}\left(1-n\right)\right]+I_{V}\left(t\right)\left[\left(-D_{r}^{\prime }\right)n+\left(1-n\right)\left(-1-C_{v}\right)\right]}{V\left(t\right)+I_{V}\left(t\right)},$$

$$x^{-}=\frac{S\left(t\right)\left(1+D_{g}^{\prime }\right)n-I_{S}\left(t\right)\left(1-n\right)}{S\left(t\right)+I_{S}\left(t\right)}.$$

Jacobian of this system can be written as follows:

$$J\left(x^{*},\;n^{*}\right)=\left(\begin{array}{cc} m\left(1-2x^{*}\right)\left[x^{+}-x^{-}\right] & mx^{*}\left(1-x^{*}\right)\left[\frac{V^{*}\left(1+C_{v}\right)+I_{V}^{*}\left(-D_{r}^{\prime }+1+C_{v}\right)}{V^{*}+I_{V}^{*}}-\frac{S^{*}\left(1+D_{g}^{\prime }\right)+I_{S}^{*}}{S^{*}+I_{S}^{*}}\right]\\ \epsilon n^{*}\left(1-n^{*}\right)\left[\theta +\omega \right] & \epsilon \left(1-2n^{*}\right)\left[\theta x^{*}-\omega \left(1-x^{*}\right)\right] \end{array}\right).$$

By setting the derivatives of x and n to zero, we can identify several fixed points for the system.

(1) For ${(x}^{*},n^{*})=(0,0)$ , we have the following form of the Jacobian:

$$J\left(0,0\right)=\left(\begin{array}{cc} m\left[\frac{-V^{*}\left(C_{v}\right)-I_{V}^{*}\left(1+C_{v}\right)}{V^{*}+I_{V}^{*}}+\frac{I_{S}^{*}}{S^{*}+I_{S}^{*}}\right] & 0\\ 0 & -\epsilon \omega \end{array}\right).$$

Eigenvalues are $\lambda_{1}=m\left[\frac{{-V}^{*}\left(C_{v}\right)-I_{V}^{*}(1+C_{v})}{V^{*}+I_{V}^{*}}+\frac{I_{S}^{*}}{S^{*}+I_{S}^{*}}\right]$ and $\lambda_{2}=-\epsilon \omega <0$ . If $\frac{I_{S}^{*}}{S^{*}+I_{S}^{*}}>\;\frac{V^{*}\left(C_{v}\right)+I_{V}^{*}\left(1+C_{v}\right)}{V^{*}+I_{V}^{*}}$ , then the fixed point is a saddle point. Otherwise, if $\frac{I_{S}^{*}}{S^{*}+I_{S}^{*}}<\;\frac{V^{*}\left(C_{v}\right)+I_{V}^{*}\left(1+C_{v}\right)}{V^{*}+I_{V}^{*}}$ , then the fixed point is stable.

(2) For ${(x}^{*},n^{*})=(1,0)$ , we have the following form of the Jacobian:

$$J\left(1,0\right)=\left(\begin{array}{cc} -m\left[\frac{-V^{*}\left(C_{v}\right)-I_{V}^{*}\left(1+C_{v}\right)}{V^{*}+I_{V}^{*}}+\frac{I_{S}^{*}}{S^{*}+I_{S}^{*}}\right] & 0\\ 0 & \epsilon \theta \end{array}\right).$$

Eigenvalues are $\lambda_{1}=\;m\left[\frac{V^{*}\left(C_{v}\right)+I_{V}^{*}\left(1+C_{v}\right)}{V^{*}+I_{V}^{*}}+\frac{I_{S}^{*}}{S^{*}+I_{S}^{*}}\right]>0$ and $\lambda_{2}=\epsilon \theta >0$ .

The fixed point is unstable equilibrium since both the eigenvalues are positive.

(3) For ${(x}^{*},n^{*})=(0,1)$ , we have the following form of the Jacobian:

$$J\left(1,0\right)=\left(\begin{array}{cc} m\left[\frac{V^{*}+I_{V}^{*}\left(-D_{r}^{\prime }\right)}{V^{*}+I_{V}^{*}}-\frac{S^{*}\left(1+D_{g}^{\prime }\right)}{S^{*}+I_{S}^{*}}\right] & 0\\ 0 & \epsilon \omega \end{array}\right).$$

Eigenvalues are $\lambda_{1}=\;m\left[\frac{V^{*}-I_{V}^{*}D_{r}^{\prime }}{V^{*}+I_{V}^{*}}-\frac{S^{*}\left(1+D_{g}^{\prime }\right)}{S^{*}+I_{S}^{*}}\right]$ and $\lambda_{2}=\epsilon \omega >0$ . If $\frac{V^{*}-I_{V}^{*}D_{r}^{\prime }}{V^{*}+I_{V}^{*}}>\frac{S^{*}\left(1+D_{g}^{\prime }\right)}{S^{*}+I_{S}^{*}}$ , then the fixed point is unstable equilibrium. If $\frac{V^{*}-I_{V}^{*}D_{r}^{\prime }}{V^{*}+I_{V}^{*}}<\frac{S^{*}\left(1+D_{g}^{\prime }\right)}{S^{*}+I_{S}^{*}}$ , the fixed point is a saddle point.

(4) For ${(x}^{*},n^{*})=(1,1)$ , we have the following form of the Jacobian:

$$J\left(1,1\right)=\left(\begin{array}{cc} -m\left[\frac{V^{*}-I_{V}^{*}D_{r}^{\prime }}{V^{*}+I_{V}^{*}}-\frac{S^{*}\left(1+D_{g}^{\prime }\right)}{S^{*}+I_{S}^{*}}\right] & 0\\ 0 & -\epsilon \theta \end{array}\right).$$

Eigenvalues are $\lambda_{1}=\;m\left[\frac{S^{*}\left(1+D_{g}^{\prime }\right)}{S^{*}+I_{S}^{*}}-\frac{V^{*}-I_{V}^{*}D_{r}^{\prime }}{V^{*}+I_{V}^{*}}\right]$ and $\lambda_{2}=-\epsilon \theta <0$ . If $\frac{S^{*}\left(1+D_{g}^{\prime }\right)}{S^{*}+I_{S}^{*}}>\frac{V^{*}-I_{V}^{*}D_{r}^{\prime }}{V^{*}+I_{V}^{*}}$ , then the fixed point is a saddle point. If $\frac{S^{*}\left(1+D_{g}^{\prime }\right)}{S^{*}+I_{S}^{*}}<\frac{V^{*}-I_{V}^{*}D_{r}^{\prime }}{V^{*}+I_{V}^{*}}$ , the fixed point is a stable equilibrium.

(5) Interior fixed point

$$\left(x^{*},\;n^{*}\right)=\left(\frac{\omega }{\omega +\theta },\frac{I_{V}^{*}S^{*}-I_{S}^{*}V^{*}+C_{v}\left(I_{S}^{*}+S^{*}\right)\left(I_{V}^{*}+V^{*}\right)}{-D_{r}^{\prime }I_{V}^{*}\left(I_{S}^{*}+S^{*}\right)-D_{g}^{\prime }S^{*}\left(I_{V}^{*}+V^{*}\right)+C_{v}\left(I_{S}^{*}+S^{*}\right)\left(I_{V}^{*}+V^{*}\right)}\;\right).$$

The Jacobian is of the form,

$$J\left(x^{*},\;n^{*}\right)=\left(\begin{array}{cc} 0 & \frac{m\omega \theta \left(-D_{r}^{\prime }I_{V}^{*}\left(S^{*}+I_{S}^{*}\right)-D_{g}^{\prime }S^{*}\left(V^{*}+I_{V}^{*}\right)+C_{v}\left(S^{*}+I_{S}^{*}\right)\left(V^{*}+I_{V}^{*}\right)\right)}{\left(S^{*}+I_{S}^{*}\right)\left(V^{*}+I_{V}^{*}\right){\left(\omega +\theta \right)}^{2}}\\ \frac{\epsilon n\left(\omega +\theta \right)\left(I_{V}^{*}S^{*}+D_{r}^{\prime }I_{V}^{*}\left(S^{*}+I_{S}^{*}\right)-I_{S}^{*}V^{*}+D_{g}^{\prime }S^{*}\left(V^{*}+I_{V}^{*}\right)\right)}{D_{r}^{\prime }I_{V}^{*}\left(S^{*}+I_{S}^{*}\right)+D_{g}^{\prime }S^{*}\left(V^{*}+I_{V}^{*}\right)-C_{v}\left(S^{*}+I_{S}^{*}\right)\left(V^{*}+I_{V}^{*}\right)} & 0 \end{array}\right).$$

If we interpret the complex matrix denoted by $\left(\begin{array}{cc} A & B\\ C & D \end{array}\right)$ as described above, where the sum of the diagonal elements A+D equals 0, and its determinant is,

$$AD-BC=\frac{m\epsilon n\omega \theta \left(\left(1+D_{g}^{\prime }\right)I_{V}^{*}S^{*}+D_{r}^{\prime }I_{V}^{*}\left(I_{S}^{*}+S^{*}\right)-I_{S}^{*}V^{*}+D_{g}^{\prime }S^{*}V^{*}\right)}{\left(I_{S}^{*}+S^{*}\right)\left(I_{V}^{*}+V^{*}\right)\left(\omega +\theta \right)}.$$

Here, the trace, A+D=0 . Thus,

(i) If the determinant (AD−BC>0) is positive, thus, $\left(1+D_{g}^{\prime }\right)I_{V}^{*}S^{*}+D_{r}^{\prime }I_{V}^{*}\left(I_{S}^{*}+S^{*}\right)+D_{g}^{\prime }S^{*}V^{*}>I_{S}^{*}V^{*}$ , the eigenvalues are real and opposite in sign, one positive and one negative, indicating a saddle point, which is not a stable equilibrium.

(ii) If the determinant (AD−BC<0) is negative, thus, $\left(1+D_{g}^{\prime }\right)I_{V}^{*}S^{*}+D_{r}^{\prime }I_{V}^{*}\left(I_{S}^{*}+S^{*}\right)-+D_{g}^{\prime }S^{*}V^{*}<I_{S}^{*}V^{*}$ , then the determinant ( AD-BC) will be negative. The eigenvalues are complex conjugates with zero real parts, suggesting that the system is marginally stable but not stable in the traditional sense. The trajectories near the fixed point neither diverge nor converge; instead, they oscillate indefinitely around the equilibrium point.
